# Effect of finishing and polishing systems on the surface roughness and color change of composite resins

**DOI:** 10.4317/jced.58011

**Published:** 2021-05-01

**Authors:** Numan Aydın, Fulya-Toksoy Topçu, Serpil Karaoğlanoğlu, Elif-Aybala Oktay, Uğur Erdemir

**Affiliations:** 1 University of Health Sciences, Gulhane Faculty of Dentistry, Department of Restorative Dental Treatment, Ankara, Turkey; 2 Istanbul University, Faculty of Dentistry, Department of Restorative Dental Treatment, Istanbul, Turkey

## Abstract

**Background:**

This study is to examine the surface roughness and color changes occurring on composite resins following the application of different finishing and polishing systems.

**Material and Methods:**

In our study, a total of 200 samples were prepared from composites resin (6×2 mm) containing supra-nano, submicron hybrid, nanohybrid, nano-ceramic and microhybrid filler. They were polished with diamond, aluminum oxide, silicon carbide finishing and polishing systems. The initial color values of composite samples were measured with a spectrophotometer and surface roughness values with a profilometer. After that, samples were immersed of coffee solution and color measurements were repeated on the 1st and 7th day. The differences between surface roughness (Ra) and color change values (∆Eab) were evaluated using two-way analysis of variance (ANOVA) test (*p*<0.05).

**Results:**

Finishing and polishing systems produced a statistically significant difference between the surface roughness values of nano-ceramic, submicron hybrid and microhybrid composite resin samples (*p*<0.05). Supra nano composite, which showed the lowest surface roughness after all finishing and polishing systems, showed the least color change after 7 days compared to other composite groups (*p*<0.05). The microhybrid composite with the highest surface roughness was the most color changing composite group (*p*<0.5).

**Conclusions:**

The spiral finishing and polishing system containing diamond particles was the system to provide the least color difference on all composite groups. However, color differences of all composite resin groups were found to exceed the perceptibility threshold (PT) and acceptability threshold (AT).

** Key words:**Composite resin, color stability, surface roughness.

## Introduction

Today, composite resins of different particle sizes, developed through nanotechnology studies dedicated to dentistry have been commonly used for the treatment of anterior and posterior teeth (1). Thanks to the development of nanofiller technology in dentistry, the esthetic properties of microfill composite resins and the mechanical properties of hybrid composite resins have been brought together and ‘nanohybrid’ composite resins have been introduced to the market. These composites consist of nanofillers (nanomer) and nanomer groups (nanocluster), and they are known to show better esthetic properties (2). It is stated that composite resins containing nano filler provide more effective color harmony with dental tissues because of their “chameleon effect” properties.

Characteristic features of a composite restorative material such as surface roughness, gloss, translucency, and color stability determine the esthetic appearance of the teeth restored with that composite (2-4). Bollen *et al.* (5) stated that surface roughness values over 0.2 µm constituted a bacterial plaque retention area.

Optical and mechanical profilometers and devices such as AFM (atomic force microscope) and SEM (scanning electron microscope) are widely used to measure and evaluate the surface roughness of restorative materials (6). Mechanical profilometers have been preferred for many years by dental experts as they require no preparation on samples to measure surface roughness, and they enable repeated measurements (7,8).

Color changes on composite resins have been associated with water absorption, chemical reaction, diet and smoking habits, poor oral hygiene, and surface roughness of the restoration. It is stated in the literature that as well as the composition of restorations, particle properties, and finishing and polishing procedures are the factors that determine the surface smoothness and susceptibility to discoloration due to external factors (9,10). Many studies have reported that beverages such as coffee, tea, cola and red wine cause discoloration on composite resin restorations at varying degrees (11-13).

Color stability of dental restorations is assessed through both visual and instrumental techniques. Instruments such as colorimeters, spectrophotometers, or digital cameras are utilized to measure color changes (ΔE*) by referring to the standards of Commission Internationale de L’éclairage (CIE) system (14,15). (L*) value indicates how light or dark the color is, (a*) refers to the redness or greenness while (b*) represents the degree of yellowness or blueness. They are also important for evaluating optical properties and should be recorded to simulate the long-term success of esthetic restorations (16).

Recently, composite resins containing different sizes of fillers are expected to be highly resistant to discoloration after finishing and polishing systems. Studies have been carried out on composite resins surface roughness of finishing and polishing systems in the literature. However, studies examining the surface roughness and color change of composite resins containing nanofiller of finishing and polishing systems of different contents are limited.

The purpose of our study was to examine surface roughness and color change of supra-nano, submicron hybrid, nanohybrid, nano-ceramic and microhybrid filler composites after different finishing and polishing applications. The first null hypothesis was that finishing and polishing systems would not create differing levels of roughness on the surface of composite resins. The second null hypothesis was that the color change of composite resins would not differ depending on the applied finishing and polishing systems.

## Material and Methods

In this study, surface roughness and color change of nano-ceramic (Ceram.x Duo, Dentsply Srona, Almanya), nanohybrid (Kerr Corporation, USA), supra-nano (Estelite Asteria, Tokuyama, Tokyo, Japan), submicron hybrid (Brilliant EverGlow, Coltene/Whaledent AG, Switzerland) and microhybrid (Amaris, Voco GmbH, Germany) composites resins (Table 1) were examined after applying Sof-Lex (3M ESPE, St. Paul, MN, USA), OptiDisc (Kerr Corporation, USA), Clearfil Twist Dia (Kuraray Noritake Dental Inc., Tokyo, Japan) and Super Snap (Shofu Inc., Kyoto, Japan) finishing and polishing systems (Table 2).

**Table 1:**
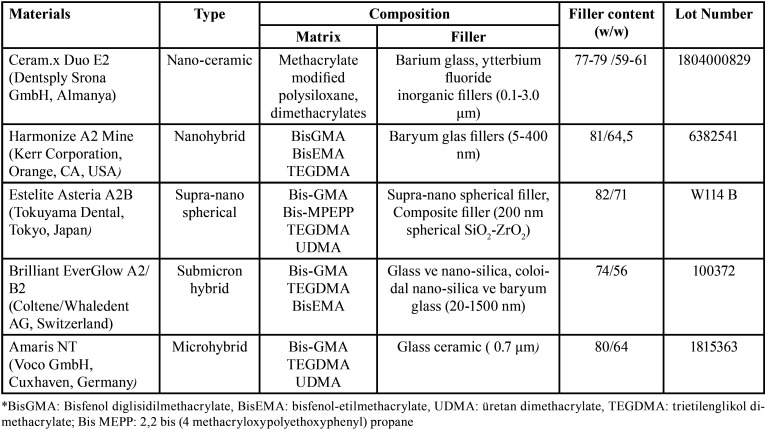
Properties of composite resin materials used in the study.

**Table 2:**
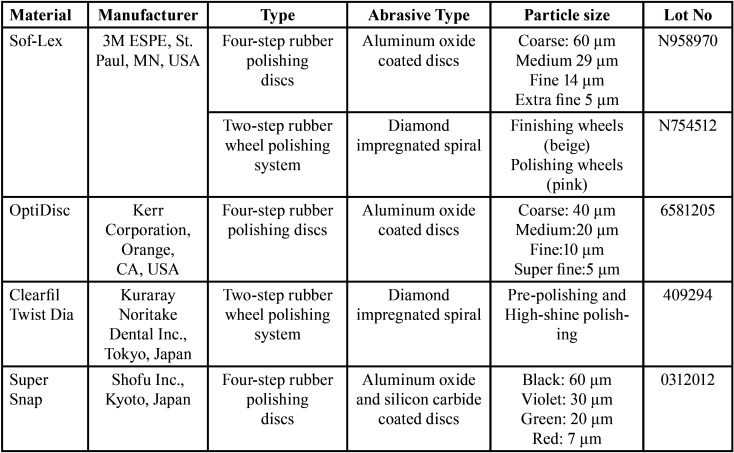
Properties of finishing and polishing discs and spirals.

-Preparation of the samples

In our study, using the analysis package program (G * Power 3.1; Universität Düsseldorf), 0.05 significance and sample size at 80% power level were calculated. After calculating the total sample size, composite resin samples (6 mm in diameter and 2 mm in height) were prepared using a metal mold for surface roughness, color change (n: 200).

Composite resins were placed in the hole on the metal mold with a mouth spatula and a 1 mm glass (coverslip) was placed on the mylar strip. Then, the samples were polymerized by touching the coverslip with the led light device (D- Light Pro, GC, Tokyo, Japan) at 1400 mW/cm² power for 20 seconds. It was divided into subgroups (n:8) for finishing and polishing composite samples. Finishing and polishing of composite resin samples was carried out for 20 seconds under water cooling with planned finishing and polishing systems (Table 2). A group of mylar strip from each different type of composite was separated as a control group.

-Surface roughness and color measurements of the composite resin samples

After finishing and polishing process, composite samples were placed into 2 ml eppendorf tubes individually and incubated in an oven (FN 500, Nüve, Turkey) in 37°C distilled water for 24 hours. Later, the initial color values (L*, a* and b*) of the samples in each group were measured with a spectrophotometer device (Vita Easyshade Advance, VITA Zahnfabrik, Germany) under D65 lighting conditions; to measure surface roughness values a profilometer device (Perthometer M2, Mahr GmbH, Germany) was used. The surface roughness and color measurements of composite samples were made from the center point of the same sample. In the measurement of the surface roughness values of the samples, the measurement length was taken as 1.75 mm and the cut-off value as 0.25. The average of these values was calculated by performing three measurements from the surface of each sample.

After determining the initial color and surface roughness values, the samples were placed into separate eppendorf tubes. A coffee solution was prepared by dissolving 2 g of coffee powder (Nescafe Classic, Nestle, Turkey) in 200 ml of boiled distilled water according to the manufacturer’s recommendation. Then, the tubes were filled with 2 ml coffee solution at 37 °C. All were incubated at 37 °C in an oven (FN 500, Nüve, Turkey) for 7 days, by refreshing coffee solutions every 24 hours. Color values (L *, a * and b *) of the samples at the end of the 1st and 7 th day were measured using the same spectrophotometer device. The amount of discolorations occurring on composite resin samples was calculated using the CIELAB color difference formula (ΔEab = [(L2-L1)2 + (a2-a1)2 + (b2-b1)2 ]1/2).

Acceptability and perceptibility thresholds (AT/PT) for discolorations are important factors to assess the color stability of restorative composite materials. In our study, in line with the literature (17), the 50:50% PT was taken as ΔEab:1.2, and the 50:50% AT was ΔEab:2.7 

-Statistical analysis

Statistical analysis of the findings was performed using the SPSS 22.00 (Statistical Package for Social Sciences, IBM Inc., USA) program. Differences between surface roughness and color change values of composite resin samples were assessed by two-way analysis of variance (ANOVA) and Tukey post hoc test was used for the differences between groups. Pearson Correlation analysis was used between the surface roughness and color change values of composite samples.

## Results

There was a statistically significant difference in Ra value formed by Sof-Lex, OptiDisc, Super Snap and Clearfil Twist Dia one of the finishing and polishing systems, on the surface of composite resin samples (*p*<0.05), (Table 3). Among the composite resin groups, the lowest Ra value was observed on the mylar strip (control group) that no finishing and polishing applied (*p*<0.05).

**Table 3:**
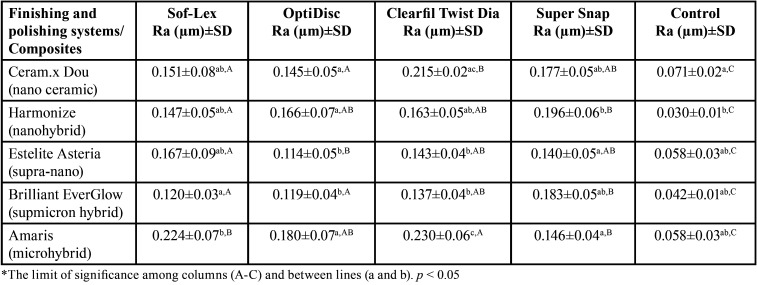
Surface roughness values (Ra) of composites which are formed by different finishing and polishing processes.

**Figure 1:**
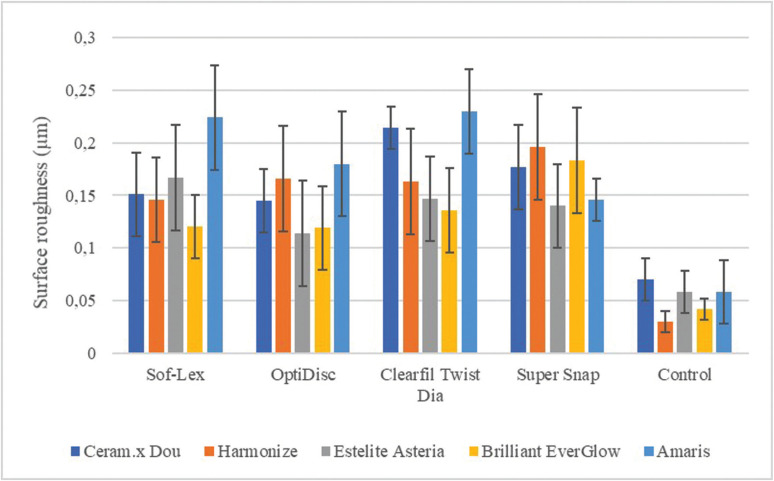
Surface roughness values (Ra) measured after the composites finished and polished.

Finishing and polishing systems experimented in the study provided significantly differing levels of effectiveness, also depending on the composite. The lowest Ra value (0.114 µm) was measured on the supra-nano composite resin (Estelite Asteria) polished with the aluminum oxide coated disc system (OptiDisc), while the highest (0.230 µm) occurred on microhybrid composite resin (Amaris) polished with the diamond spiral system (Clearfil Twist Dia), (Fig. 1).

On the 1st and 7th day after coffee immersion, there was a significant difference between the color change values of composite resins polished with different systems (*p*<0.05). At the end of 7 days, the lowest color change (∆Eab: 4.4) was observed on supra-nano composite (Estelite Asteria) while the highest (∆Eab: 12.8) was on microhybrid (Amaris) composite (Table 4).

**Table 4:**
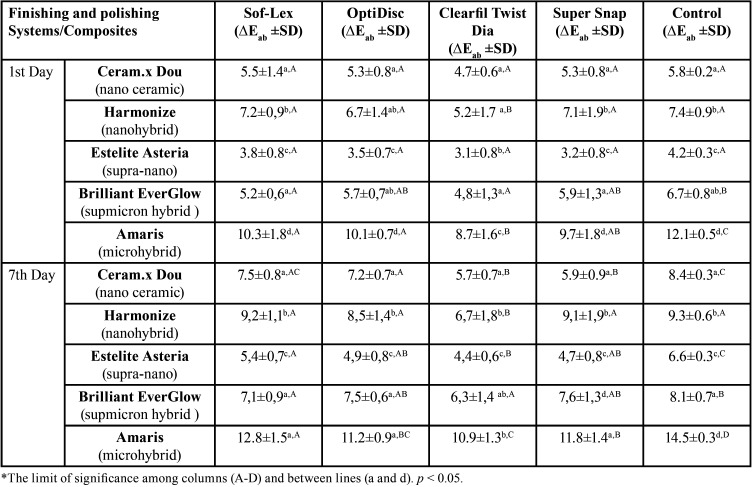
Examination of color change values (∆Eab) on the 1st and 7th days after different finishing and polishing systems of composites.

**Figure 2:**
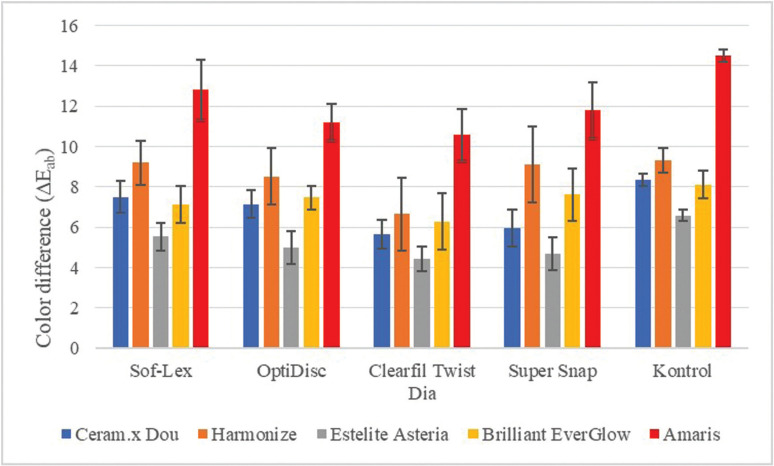
Color change values (ΔEab) on the 7th day after the composites finished and polished.

Among the all composite groups tested, the lowest color change occurred on samples polished with the diamond spiral system (Clearfil Twist Dia), while the highest discoloration was seen on the mylar strip (control) group that was not finished and polished. All finishing and polishing systems created lowest color change on supra nano composite. Color changes of all composite groups increased over time after finishing and polishing processes (Table 4, Fig. 2). In our study, the correlation analysis between the color change of the composite samples and the surface roughness values was not statistically significant (*p*>0.05).

## Discussion

It is stated that the final surface smoothness obtained by finishing and polishing processes is important in terms of esthetic appearance and mechanical properties considering the further development of mechanical and physical properties of composite resins (18,19). Low surface roughness increases the esthetic appearance and total success of composite resins, while rough surfaces cause plaque accumulation, recurrent decays, and discoloration of the restoration (20).

Various systems are used in the finishing and polishing of composite resin materials. These systems they differ greatly in their composition, presentation, type, and hardness of abrasive particles. It is stated that it is important to know which finishing and polishing systems offer adequate surface quality to increase the success of resinous restorations in clinical use (21). Therefore, in our study, we examined the surface roughness and color change on composite resins of finishing and polishing systems.

Although surface roughness of composite materials is stated to be associated with the size and content of the fillers, it is also reported to be influenced by the filler particle type, the degree of polymerization of the polymer matrix, and silane binders (22).

Yadav *et al.* (23) stated that the finishing and polishing systems examined the effect of composite resins on the surface roughness, and the lowest surface roughness of the composites tested was observed in the nanofill (Ceram x mono) composite.

In a study on the surface roughness of the composite resins, Aytac *et al.* (24) reported that the lowest value was obtained in the supra-nano composite (Estelite Ʃ Quick) and there was no significant difference between the surface roughness values of the Filtek Z250, Filtek Z550 and Clearfil Majesty ES 2 composite groups.

In our study, among the composite resin groups, the lowest surface roughness value (Ra: 0.114 µm) was measured on the supra-nano composite (Estelite Asteria) and the highest value (Ra: 0.230 µm) on the microhybrid (Amaris) composite. In our study, supra-nano composite showed lowest surface roughness similar to that of Aytac *et al.* (24) In our study, as the finishing and polishing systems produced different degrees of roughness on the surface of composite resins in our study, the first null hypothesis was rejected.

The surface roughness of composite resins can change depending on the polishing process and the structure, flexibility, hardness, and grain sizes of the materials used in these processes. In the literature, it has been reported that the lowest Ra values on the surface of composite resins are provided by aluminum oxide discs (24,25). However, as a major disadvantage of discs, it is stated that the frictional force applied during the polishing process causes cracks on the polymer matrix surface (23).

In our study, similar to some studies in the literature (24,25), the finishing and polishing system (OptiDisc) containing aluminum oxide gave the lowest surface roughness value (Ra: 0.114 µm). While the combined system in which aluminum oxide and diamond particle spiral systems are used one after the other (Sof-Lex) and the aluminum oxide and silicon carbide (Super Snap) systems exhibited close Ra values, in the diamond particle system (Clearfil Twist Dia) the highest roughness (Ra: 0.230 µm) was observed. As diamond particles are harder than aluminum oxide and silicon carbide particles, it creates a rougher surface on composite materials in finishing and polishing processes. However, the supra-nano composite did not show significantly different results in aluminum oxide or diamond particle finishing and polishing system applications, probably because it contains nanoparticles equal in size of its equivalents.

As previously stated, although there is no currently accepted threshold value in surface roughness assessment, Bollen *et al.* (5) reported that Ra values above 0.2 µm may cause an increase in plaque accumulation, an increased risk of decay, and periodontal inflammation, finishing and polishing systems tested in our study provided Ra values below 0.2 μm (except for microhybrid composite).

Discolorations occurring despite the finishing and polishing applications effectively performed on composite resins cause patient dissatisfaction and they are mostly perceived as the esthetic poverty of the materials (26). It is stated that these color changes, which cannot be accepted as ‘clinical problems’ alone, are associated with various internal and external factors such as chemical reactions, insufficient polymerization, water absorption, poor oral hygiene, and nutritional habits (27).

In the field of dentistry, the CIELAB color system is referred to in many studies to measure and assess the color changes on teeth and restorative composites. In their comprehensive review of the relevant literature, Paravina *et al.* (28) reported that CIELAB color difference formula (∆Eab) 50:50 PT (∆Eab: 1.2) and 50:50 AT (∆Eab: 2.7). In our study, all the composite samples immersed in coffee for 7 days showed color change over PT and AT values. After 7 days of coffee immersion, supra-nano composite (Estelite Ateria) showed moderately unaccepTable, nano-ceramic (Ceram.x Dou) and submicron hybrid composite (Brilliant EverGlow) clearly unaccepTable, and nanohybrid (Harmonize) and microhybrid composite (Amaris) extremely unaccepTable results in all finishing and polishing systems.

It has been reported that the structure and filler particle properties of composites have a direct effect on surface roughness and external colorations. Choi *et al.* (29) in which different composite resins were immersed in colorant solutions after finishing and polishing performed with Super-Snap, Sof-Lex and Enhance systems, no significant difference was found between the finishing and polishing systems in terms of Ra values. Nevertheless, the color changes on the surfaces finished with the Enhance instruments were lower.

Patel *et al.* (9) reported that among the composites finished with different systems and immersed into various colorant solutions (coffee, red wine, and cola), the surfaces showing the highest color change were obtained in the groups under the mylar strip. Therefore, they suggested that the resin-rich layer must be removed for the sake of color stability.

By examining discolorations of esthetic restorative materials finished and polished with different systems, and immersed into coffee for 28 days, Beltrami *et al.* stated that the lowest color change was observed on nanofill composites, which was followed by nanohybrid and microhybrid composites (30). They also concluded that as the composite particle size decreases, discolorations would also diminish depending on the lowering surface roughness.

Topcu *et al.* (31) in their study on the effects of different beverages (lemon juice, coffee (unsweetened), cola, cherry juice, fresh carrot juice, and red wine) on the color stability of composite resins, stated that micro-hybrid composite (Filtek Z250) showed a higher level of color change than the nanofill composite (Filtek Ultimate), and the fact that the nanofill composite exhibited a lower color change depends on the nanoparticle size.

 In our study, the composite resin groups, which were finished with mylar strip, were the highest discoloration groups at the end of day 1st and 7th, similar to Patel *et al.* (9). In comparison according to the finishing and polishing systems, the lowest color change was detected on the samples finished with the diamond spiral system (Clearfil Twist Dia). On the surface of nano-ceramic, supra-nano, nanohybrid and microhybrid composite samples, the diamond spiral system (Clearfil Twist Dia) produced the lowest color change, while aluminum oxide coated disk and the diamond-containing spiral system (Sof-Lex) produced the highest color change.

Yikilgan *et al.* (32) stated in their study that there was no correlation between surface roughness and color change. In our study, there was no correlation between the surface roughness of composites and color changes.

In terms of the color stability of composites, material content and the structure of the resin matrix play an important role as well as the finishing and polishing systems. Diyetschi *et al.* (33) stated that the discolorations on composite resin restorations are occurring due to intolerable water absorption caused by the high resin content. Also, it has been reported that as Bis-GMA involving in the organic matrix causes rigid network formation, the composites whose main monomer content is Bis-GMA tend to show less water absorption than the ones containing TEGDMA; however they are relatively more susceptible to water absorption than the composites containing UDMA and Bis-EMA (34). In their study on the water absorption of monomers in composites, solubility and discoloration, Fonseca *et al.* (35) found that in terms of absorption, solubility, and color change monomers were listed as BisEMA <UDMA <BisGMA.

Relevant studies in the literature reported that the matrix structure is significantly associated with the color change of composite resins and the highest color change is caused by the TEGDMA monomer (29,36). In our study, among the composites with similar monomer content (BisGMA, UDMA and TEGDMA), supra-nano composite resin (Estelite Asteria) exhibited the lowest color change (∆Eab: 4.4), while micro-hybrid composite resin (Amaris) showed the highest color change (∆Eab: 12.8). Apart from this, the submicron hybrid composite (Brilliant EverGlow,) with the same monomer content (BisGMA, BisEMA and TEGDMA) showed lower color change than the nanohybrid composite (Harmonize). Therefore, the main reason for the color changes on the composites we tested in our study is thought to be the particle size, type, and dispersion rather than the organic monomer content.

In our study, after the finishing and polishing system, the composites showed color change above the levels of acceptability threshold (ΔEab: 2.7). However, this study is an *in vitro* study that induces stains on both sides of the restorative material. In the clinical situation, the material is attached to a tooth structure (enamel or dentin), and only its surface is exposed to solutions. In addition, the drinks included in the diet cause more external discoloration on resin composites. The rate at which these color changes in composite resins can be removed by after polishing or bleaching can be discussed in future research.

## Conclusions

According to the results of our *in vitro* experiment examining the surface roughness and color change of composite resins after applying different finishing and polishing systems.

1. Finishing and polishing systems create different surface roughness according to the particle size of composite resins.

2. All finishing and polishing systems created roughness below 0.2 μm on the surfaces of the composite samples (except for microhybrid composite).

3. As the composite resin particle size decreases, finishing and polishing systems create less surface roughness and discoloration.

4. In our study, finishing and polishing systems containing diamond particles on all composite resins groups had less color variation.

5. After different finishing and polishing systems, all composite resin groups showed color difference above perceptibility threshold (ΔEab: 1.2) and acceptability threshold (ΔEab: 2.7) values.
